# First person – Julia Loose

**DOI:** 10.1242/bio.058773

**Published:** 2021-05-07

## Abstract

First Person is a series of interviews with the first authors of a selection of papers published in Biology Open, helping early-career researchers promote themselves alongside their papers. Julia Loose is first author on ‘ Auxin treatment increases lifespan in *Caenorhabditis elegans*’, published in BiO. Julia is a PhD student in the lab of Arjumand Ghazi, PhD at the University of Pittsburgh Rangos Research Center, Pittsburgh, USA, investigating the relationship between reproduction and aging.


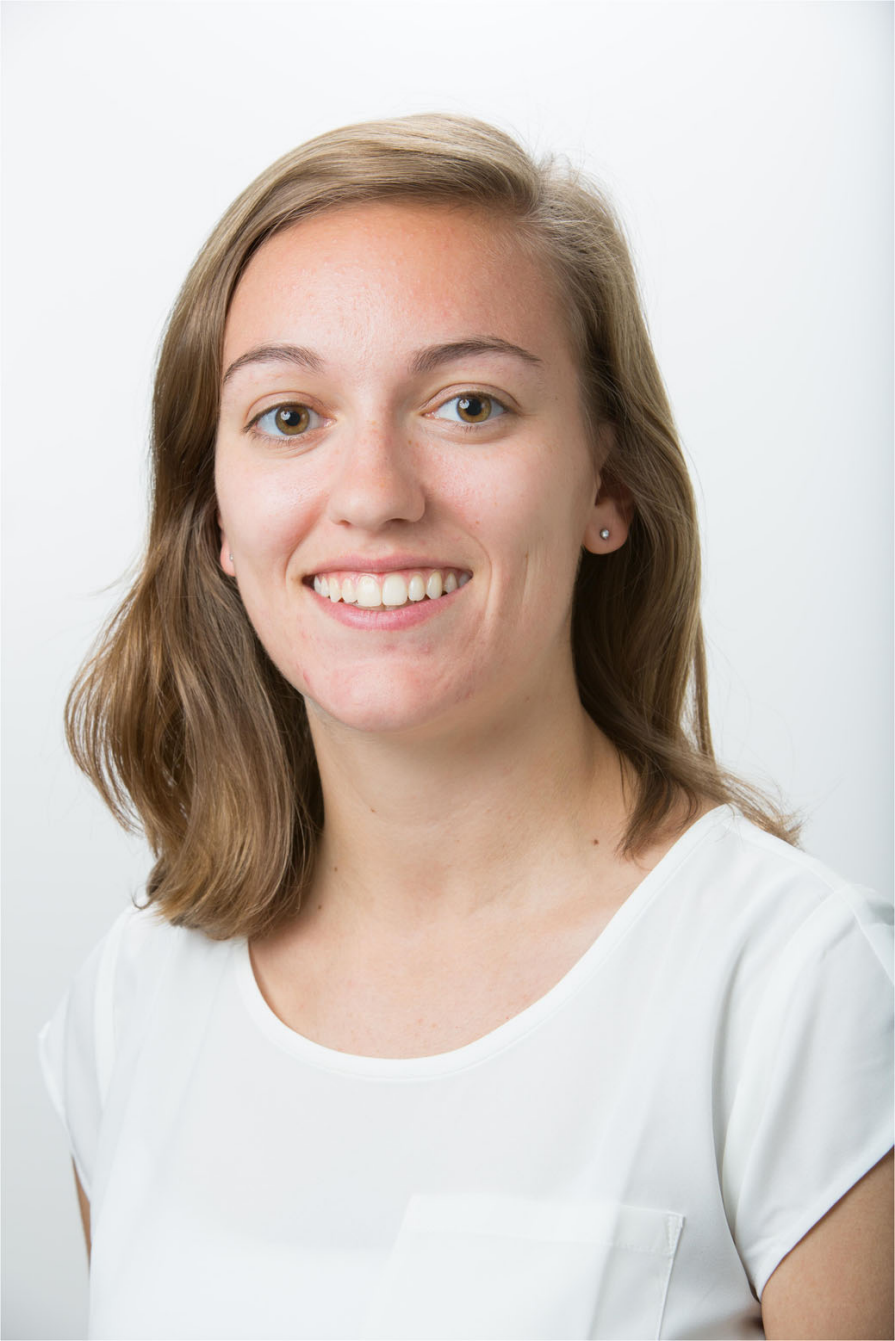


**Julia Loose**

**What is your scientific background and the general focus of your lab?**

Currently I am completing my PhD in Molecular Genetics and Developmental Biology in the lab of Arjumand Ghazi at the University of Pittsburgh. Before entering graduate school I explored many different areas of biology through summer research experiences including studying calcium transport during development as well as using checkpoint inhibitors to rescue oocyte loss during radiation. I joined the Ghazi lab for my PhD research with an interest in reproductive biology. The main focus of the Ghazi lab is understanding the molecular basis for aging. We mainly use the model organism *Caenorhabditis elegans* to study these questions which has led to different areas of research including the relationship between reproduction and aging.

**Figure:**
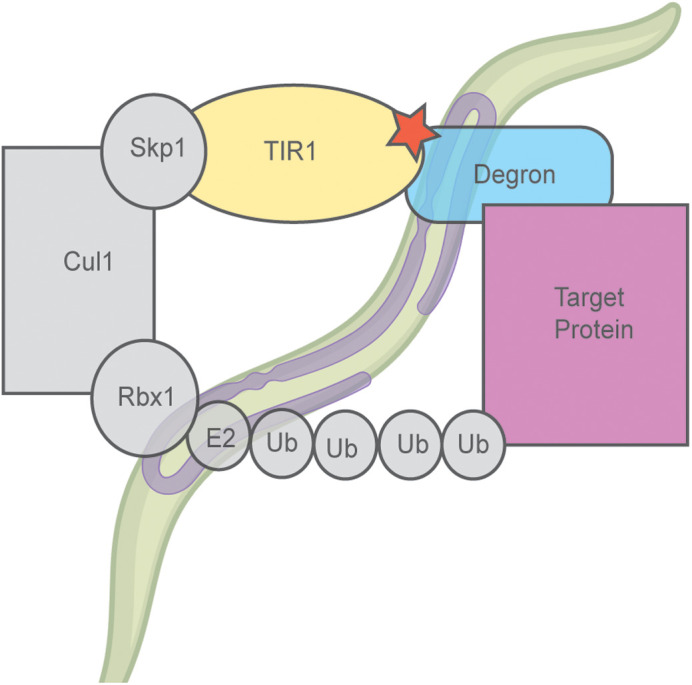
Schematic representation of the auxin degradation system.

**How would you explain the main findings of your paper to non-scientific family and friends?**

In order to further understand the function or role of a gene in an organism, researchers often study what happens when the gene or the protein resulting from the gene's expression, is disrupted. Many different tools have been developed to either mutate the gene so the gene is not expressed, or knockdown the protein so that the protein cannot function in the organism. Recently a tool was developed in the model organism, *C. elegans* that results in knockdown of a specific protein when the animal is exposed to the chemical, auxin. We found that this tool and the methods developed around this tool had other effects on the organisms in addition to just knockdown of the protein. Specifically, we found that exposure of *C. elegans* to auxin increased their lifespan. This result is important for other researchers who plan to use these tools, specifically for those studying aging.

**What are the potential implications of these results for your field of research?**

The results we have presented in our paper emphasize to researchers the importance of assessing different aspects and sometimes unintended consequences of experimental technologies. *C. elegans* are a useful tool for studying the genetic basis of aging and new technologies have helped to advance the aging field forward.

**What has surprised you the most while conducting your research?**

I was most surprised when we found that exposing wild-type *C. elegans* to auxin influenced their lifespan. This was an unexpected result that we observed when we initially were studying the impact of knocking down proteins involved in reproduction. However upon further replicates we found that this observation repeated in other trials. I think overall in research it is surprising when an experiment aims to answer one question, but the results give an answer to a question we did not intend to even ask.

“I think overall in research it is surprising when an experiment aims to answer one question, but the results give an answer to a question we did not intend to even ask.”
